# Overview of polymyxin resistance in Enterobacteriaceae

**DOI:** 10.1590/0037-8682-0349-2021

**Published:** 2022-02-25

**Authors:** Kesia Esther da Silva, Luana Rossato, Andressa Ferraz Leite, Simone Simionatto

**Affiliations:** 1 Universidade Federal da Grande Dourados, Laboratório de Pesquisa em Ciências da Saúde, Dourados, MS, Brasil.; 2 Stanford University, Division of Infectious Diseases and Geographic Medicine, Stanford, CA, USA.

**Keywords:** Enterobacteria, Polymyxin resistance, Molecular mechanism, Epidemiology

## Abstract

Polymyxin antibiotics are disfavored owing to their potential clinical toxicity, especially nephrotoxicity. However, the dry antibiotic development pipeline, together with the increasing global prevalence of infections caused by multidrug-resistant (MDR) gram-negative bacteria, have renewed clinical interest in these polypeptide antibiotics. This review highlights the current information regarding the mechanisms of resistance to polymyxins and their molecular epidemiology. Knowledge of the resistance mechanisms and epidemiology of these pathogens is critical for the development of novel antibacterial agents and rapid treatment choices.

## INTRODUCTION

Polymyxins have been used for over 50 years in both veterinary and human medicine. Colistin is a decapeptide administered either as colistin sulfate, an oral prodrug, or as colistin methanesulfonate (CMS) when used intravenously^1^. There are five types of polymyxins, from A to E, but only colistin (also known as polymyxin E) and polymyxin B were clinically used in the 1950s, as they were found to be the least nephrotoxic^2^. Ultimately, these antibiotics fell out of favor, and their systemic use was reduced due to their considerable adverse effects, particularly their potential for nephrotoxicity and neurotoxicity^3^. Attempts to reduce nephrotoxicity through dosing have also been discussed^4^ ^,^ ^5^. Among these, extensive monitoring of renal function during therapy, avoiding the co-administration of other known nephrotoxic agents when possible, and maintaining an adequate fluid and electrolyte balance are essential components of approaches that may reduce the risk of polymyxin-associated acute kidney injury^5^. However, interest in systemic polymyxins has recently reignited owing to the growing incidence of infections caused by multidrug-resistant (MDR) gram-negative bacteria^6^. Unfortunately, extensive use of colistin as a livestock food additive, along with its inappropriate use in clinical medicine, has led to reservoirs of high levels of resistance in gram-negative bacteria, such as *Acinetobacter baumannii,* Enterobacteriaceae (*Klebsiella pneumoniae* and *Escherichia coli*), and *Pseudomonas aeruginosa* ^7^. Although the value of polymyxins now used in health centers is acknowledged, novel derivatives that are less toxic and more effective are needed. CA824, FADDI-02, MicuRx-12, FADDI-287 and SPR206 [previously CA1206] are polymyxins derivatives shown to be superior to the old polymyxins in human clinical trials and rodent lung infection models with *P. aeruginosa* and/or *A. baumannii* ^8^ ^,^ ^9^ Despite the improvements in the discovery of new polymyxins derivatives, investigation groups also have made excellent progress in clarifying the mechanism of colistin resistance. The goal of the present review is to discuss the molecular mechanisms of polymyxin resistance and their molecular epidemiological data. 

### Mechanisms of Polymyxin Resistance

**FIGURE 1:**
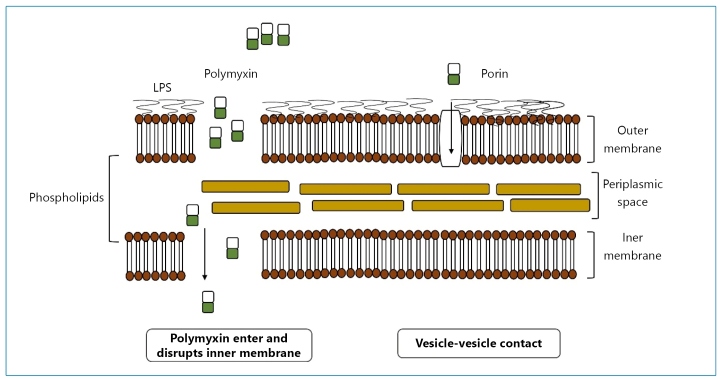
Antibacterial mechanisms of polymyxin: (a) classic mechanism of membrane lysis; (b) alternative mechanism of vesicle-vesicle contact. LPS: lipopolysaccharide22

Polymyxins are cyclic peptides that share almost identical primary structures. Polymyxin B is currently used in antimicrobial therapy^10^. Polymyxins selectively bind to lipopolysaccharides (LPSs), thereby acting on the membranes of gram-negative bacteria (Figure 1). LPSs are composed of three domains: the central oligosaccharide, lipid A, and O antigen^11^. Lipid A, the most vital domain, plays an essential role in maintaining the stability and integrity of membrane structures. Initially, polymyxins electrostatically interact with lipid A phosphate groups and replace the calcium and magnesium ions, whose function is to cross-bridge adjacent lipid A molecules and stabilize the outer membrane. These interactions result in the decline of lipid A and the subsequent disruption of the membrane, leading to cell lysis and death^12^.

Some chromosomal mutations have been associated with colistin resistance, as they lead to changes in the outer membrane elements essential to polymyxin function. Polymyxin resistance is mediated mainly by the structural modification of membrane LPSs through regulatory systems. These changes can reduce the electrostatic attraction between the phosphate groups of lipid A and polymyxin molecule^13^ ^,^ ^14^.

Modification of the chemical composition of lipid A via the biosynthesis and addition of phosphoethanolamine (pEtN) and 4-amino-4-deoxy-L-arabinose (L-Ara4N) are the most common mechanisms. The master regulator of polymyxin resistance includes a two-component Pho-PQ system. Sub-lethal concentrations of polymyxin induce the PhoQ kinase sensor to phosphorylate PhoP, leading to activation of the PmrA-PmrB system via the PmrD protein^15^. Therefore, the PmrA-PmrB system stimulates the expression of the arnBCADTEF operon, which is necessary for the covalent alteration of the phosphate groups in lipid A. Structural modifications decrease the negative charge on the membrane, avoiding interactions with polymyxin^13^.

Another regulatory system, crrAB, acts as a mediator of polymyxin resistance. It comprises a histidine kinase (crrB) and an inactivated or absent response regulator (crrA) in certain strains of *K. pneumoniae*, leading to activation of the pmrCAB system^16^. Numerous mutations have been documented in the genes involved in polymyxin resistance. The most common mechanism found in *K. pneumoniae* involves inactivation of mgrB through nonsense mutations, nucleotide deletion, and truncation by insertion elements. Recently, a clonal spread of polymyxin-resistant *K. pneumoniae* isolates was described for the first time, with polymyxin resistance linked with various changes in the *mgr*B gene involving inactivation by an insertion sequence and nonsense point mutations. The results showed that *mgr*B alterations were the most frequent source of polymyxin resistance in Brazilian clinical settings^17^. 

Regarding inactivation by insertion elements (ISs), the IS5 family is the most frequently found, followed by the IS1 family. These ISs can be inserted into the coding or promoter regions of the gene^18^ ^,^ ^19^. Lipid A content can also be altered by the addition of pEtN. This is the most important resistance mechanism observed in *A. baumannii* and may involve several genes, including eptB (pagC), eptA (pmrC), and eptC (cptA)^16^. 

The loss of the O-antigen through the mutation of genes implicated in the biosynthesis of this component has already been delineated in *Yersinia enterocolitica*, *Salmonella* spp., and Enterobacteria. Reduced susceptibility to polymyxin may be attributed to other regulatory genes that modulate lipid A biosynthesis, such as ramA^20^ ^,^ ^21^.

Efflux pumps can also contribute to polymyxin resistance, and several efflux pump regulators have been observed in different species, such as BrlR, sensitive antimicrobial peptides (Sap) proteins, KpnEF, or the AcrAB-TolC complex. Generally, the activation of these pumps leads to increased resistance to several antibiotics at the same time, including polymyxin^22^. Increased expression of genes encoding capsule synthesis has also been observed in strains of *K. pneumoniae*, *E. coli*, and *P. aeruginosa*, which cause resistance to polymyxin^16^. These findings highlight the importance of bacterial capsules for polymyxin resistance.

Polymyxin resistance was initially described to be associated with chromosomal mechanisms with no possibility of horizontal transfer. In 2016, a new plasmid-mediated resistance gene was identified in bacterial isolates^23^. The *mcr-*1 gene encodes an enzyme of the phosphoethanolamine transferase family, which is responsible for the synthesis and conjugation of pEtN to lipid A. The first description occurred in China of bacterial isolates from animal foods (chickens and pigs). In humans, the first isolate was identified in Latin America as an *E. coli* strain recovered from a hospitalized patient^24^ ^-^ ^26^. To date, nine variants of the *mcr*-1 gene have been identified and sequentially named *mcr*-1 to *mcr*-9^27^. In enterobacteria, the genes *mcr*-1, *mcr*-2, and *mcr*-3 have been isolated in plasmids and recently identified in the chromosomes of *Moraxella* spp. and *Aeromonas* ^28^ ^,^ ^29^.

Plasmid-mediated horizontal transfer results in the rapid spread of resistance genes among several bacterial species, which is responsible for a wide variety of MDR phenotypes in bacteria that can cause infections in humans and animals. The existence of *mcr*-1 and other resistance genes suggests the presence of different pathways for the horizontal transmission of colistin resistance and its high potential for propagation. The *mcr*-1 variant can be connected to various types of plasmids, including IncHI2, IncI2, IncP, IncX, and IncFIP. The association of these plasmids with other genes that confer resistance has also been established, with reduced susceptibility to quinolones, cephalosporins, and fosfomycin discovered^16^ ^,^ ^30^. 

In a retrospective study, the *mcr*-1 gene was identified in *E. coli* strains obtained from chicken farms in the 1980s, the same period when colistin was introduced to China's livestock. The frequency of *mcr*-1 was found to be 20% in animal bacterial strains and 1% in human bacteria in China^23^. A few months after being reported for the first time, *mcr*-1 has been detected in bacterial isolates from animals, humans, and the environment in various countries in South America, North America, Europe, Asia, and Africa, and has been identified in several bacterial genera, including *Escherichia*, *Shigella, Klebsiella*, *Salmonella*, *Enterobacter*, and *E. coli* ^31^ ^-^ ^35^.

### Molecular Epidemiology of Polymyxin-Resistant Enterobacteria

Antimicrobial resistance is a major challenge to human and animal health in the 21st century, and polymyxin resistance appears to be an even more serious problem, compounded by the fact that an efficient policy for the use of antibiotics in animal and human production is absent in some countries. The worldwide occurrence of resistance to polymyxins is 10% among gram-negative bacteria, with higher rates in Southeast Asian and Mediterranean countries^30^ ^,^ ^36^ ^,^ ^37^. The increase in the use of polymyxin for infections caused by MDR gram-negative bacteria has led to the emergence of resistance in several countries worldwide, and its prevalence may vary among regions. Countries such as South Africa and Japan do not have access to polymyxin, and some areas of the world have only colistin formulations, while, in other areas, including Brazil, USA, Singapore, and Malaysia, clinicians prescribe parenteral formulations of colistin or polymyxin B^38^. Reports are scarce in African countries, whereas studies in South Africa and Nigeria have reported resistance rates of less than 10%^39^. In the Asian region, resistance to colistin is common mainly in isolates of *Enterobacter* spp. and *Klebsiella* spp., prevalent in all countries in the region except Singapore, with rates ranging from 13.8% (India) to 50% (Philippines)^30^ ^,^ ^36^ ^,^ ^40^. 

In Brazilian hospitals, there was an increase in the rate of polymyxin-resistant *K. pneumoniae* from 1.8% in 2009 to 15% in 2013 and 35.5% in 2015^41^. It is considered endemic and is frequently associated with high rates of morbidity and mortality in patients^17^. A study conducted in São Paulo, Brazil on KPC-producing *K. pneumoniae* isolates demonstrated that the polymyxin resistance index varied from 0% in 2011 to 27% in 2015^42^ ^-^ ^44^. Some studies carried out in different Brazilian hospitals have identified strains of polymyxin-resistant enterobacteria, whose responsible mechanisms include modification of membrane LPS through inactivation of *mgr*B^17^ and the presence of the *mcr*-1 gene^32^ ^,^ ^45^ ^,^ ^46^. Although reports of cases from the USA and Europe are generally rare, an increasing incidence from these regions has been recorded in recent years^47^ ^,^ ^48^.

The identification of polymyxin resistance genes in microorganisms isolated from animal food has rekindled debate regarding the contribution of the undiscovered use of antibiotics in animals to detect high levels of resistance in humans. Resistance to polymyxin, encoded by the *mcr*-1 gene, is believed to have been disseminated from animals to humans, based on the fact that they were primarily obtained in groups of animals that consume a large amount of this antibiotic during development. It is estimated that approximately 12,000 tons of colistin are utilized per year in food production, and that utilization is expected to rise to 16,500 tons by 2021. In view of this scenario, preventive measures need to be implemented to prevent the continuous dependence on this antibiotic and control the spread of this resistance^35^.

## CONCLUSIONS

Colistins and polymyxin B are potent bactericidal agents against enterobacteria. However, polymyxin-resistant strains have emerged at an alarming rate. As discussed in this review, it is imperative that rigorous control measures that prevent dissemination, as well as cautious use of polymyxins, are critical until new drugs or alternative therapeutic advances are available. In addition, studies of the molecular epidemiology of the distribution and dissemination of *mcr* genes should be conducted. 
